# Crystal structure of dimethyl 5-(4-ethyl­phen­yl)-4-[(4-ethyl­phen­yl)ethyn­yl]-6,11-diphenyl-1,3,6,11-tetra­hydro-2*H*-6,11-ep­oxy­cyclo­penta­[*a*]anthracene-2,2-di­carboxyl­ate

**DOI:** 10.1107/S2056989020006404

**Published:** 2020-05-29

**Authors:** Xiang-Zhen Meng, Dong Cheng

**Affiliations:** a School of Chemistry and Materials Engineering, Chaohu College, Chaohu Anhui, People’s Republic of China

**Keywords:** crystal structure, C—H⋯π inter­actions, Hirshfeld surface analysis, hexa­dehydro-Diels–Alder reaction

## Abstract

In the central fused ring system of the title compound, C_51_H_42_O_5_, all of the five-membered rings are in an envelope conformation. The dihedral angle between the two benzene rings in the fused ring system is 74.66 (7)°. In the crystal, mol­ecules are linked by C—H⋯π inter­actions, forming a layer parallel to the *ab* plane. Each mol­ecule acts as a double donor as well as a double acceptor of the C—H⋯π inter­actions. Hirshfeld surface analysis and two-dimensional fingerprint plots indicate that the most important contributions to the crystal packing are from H⋯H (61.4%) and C⋯H/H⋯C (25.3%) contacts.

## Abstract 2

In the fused ring of the title compound, C_51_H_42_O_5_, all of the five-membered rings are in an envelope conformation. The dihedral angle between the benzene rings attached to the fused ring is 74.66 (7)^*o*^.

## Chemical context   

The hexa­dehydro-Diels–Alder reaction, by which benzyne inter­mediates (Niu *et al.*, 2013 ▸) as well as highly functionalized benzenoid products (Karmakar *et al.*, 2013 ▸) are prepared, has played a very significant role in the field of organic synthesis. Zhang *et al.* (2015 ▸) observed that benzyne inter­mediates can be captured by five-membered heterocyclic compounds, such as furans, pyrroles and thio­phenes. As part of our work on the application of the hexa­dehydro-Diels–Alder reaction (Meng *et al.*, 2017 ▸), we report herein the synthesis and crystal structure of the title compound.
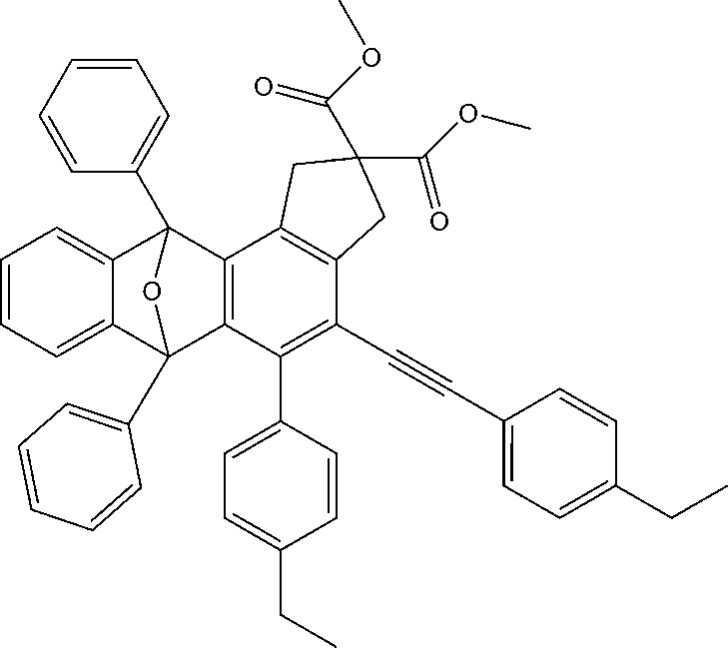


## Structural commentary   

**Figure 1:**
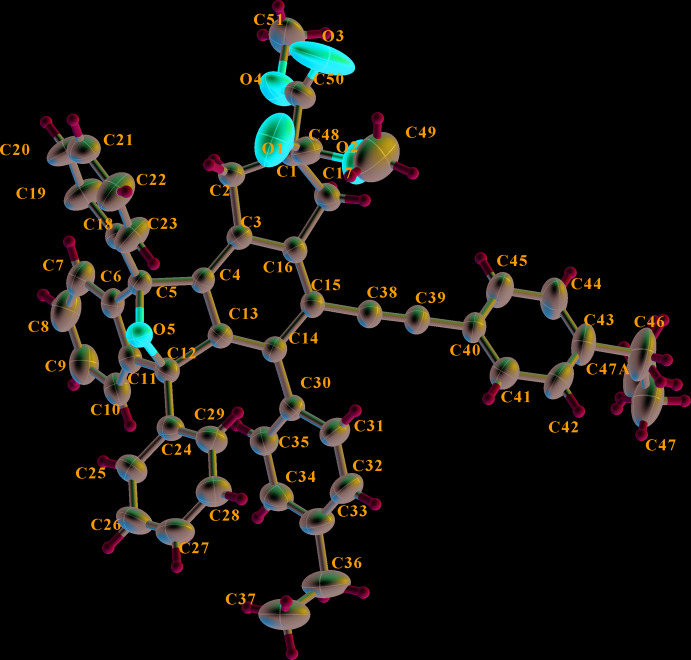
The mol­ecular structure of the title compound, with atom labels and displacement ellipsoids drawn at the 50% probability level. H atoms are shown as small circles of arbitrary radii.

The mol­ecular structure of the title compound is shown in Fig. 1 ▸. One ethyl group (C46–C47) is disordered over two sites around the C43—C46 bond axis with occupancies of 0.548 (9) and 0.452 (9). The fused ring system is not planar, and the five-membered rings (C4/C5/O5/C12/C13 and C5/C6/C11/C12/O5) adopt envelope conformations with atom O5 as the flap. The C4–C6/C11–C13 ring adopts a boat conformation. The C1–C3/C16/C17 ring adopts an envelope conformation with atom C1 as the flap and has puckering parameters *Q*_2_ = 0.3132 (18) Å and *φ*_2_ = 178.5 (3)°. The angle between the two benzene rings (C6–C11 and C3/C4/C13–C16) in the fused ring system is 74.66 (7)°. The *sp*-hybridized character of atoms C38 and C39 is confirmed by the C38—C39 [1.188 (2) Å] bond length, and the C15—C38—C39 [177.2 (2)°] and C38—C39—C40 [178.4 (2)°] bond angles. The C3/C4/C13–C16 benzene ring is inclined to the C30–C35 and C40–C45 benzene rings by 57.72 (7) and 35.48 (3)°, respectively, the latter two rings being inclined to each other by 77.78 (7)°.

## Supra­molecular features   

**Figure 2:**
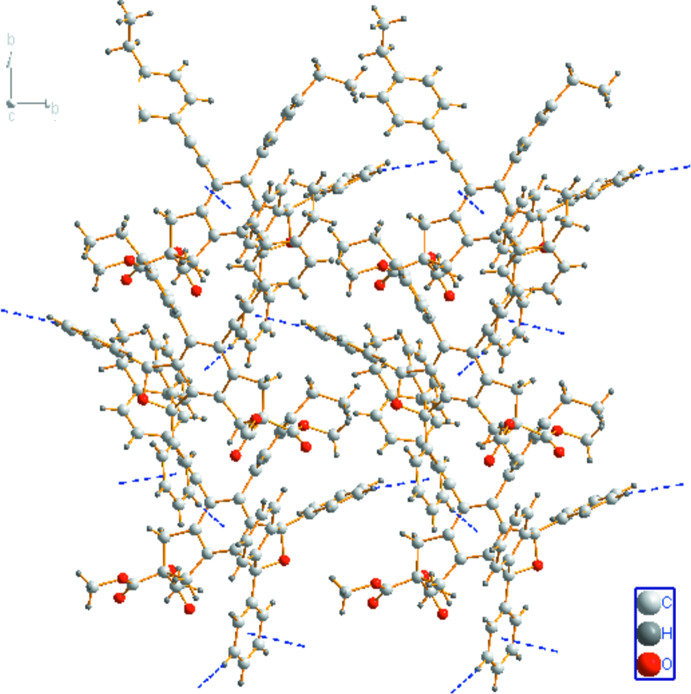
A packing diagram of the title compound, viewed along the *c* axis. The C—H⋯π inter­actions are shown as dashed lines.

In the crystal, the mol­ecules are linked by weak C—H⋯π inter­actions (C20—H20⋯*Cg*4^i^ and C26—H26⋯*Cg*7^ii^; symmetry codes as in Table 1 ▸), forming a layer parallel to the *ab* plane (Fig. 2 ▸); *Cg*4 and *Cg*7 are the centroids of the C3/C4/C13–C16 and C18—C23 rings, respectively.

**Figure 3:**
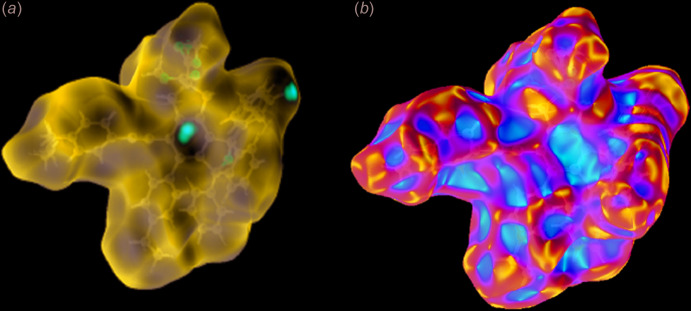
(*a*) The Hirshfeld surface mapped over *d*_norm_ in the range −0.260 (red) to 1.846 (blue) a.u., and (*b*) the Hirshfeld surface mapped over shape-index.

**Figure 4:**
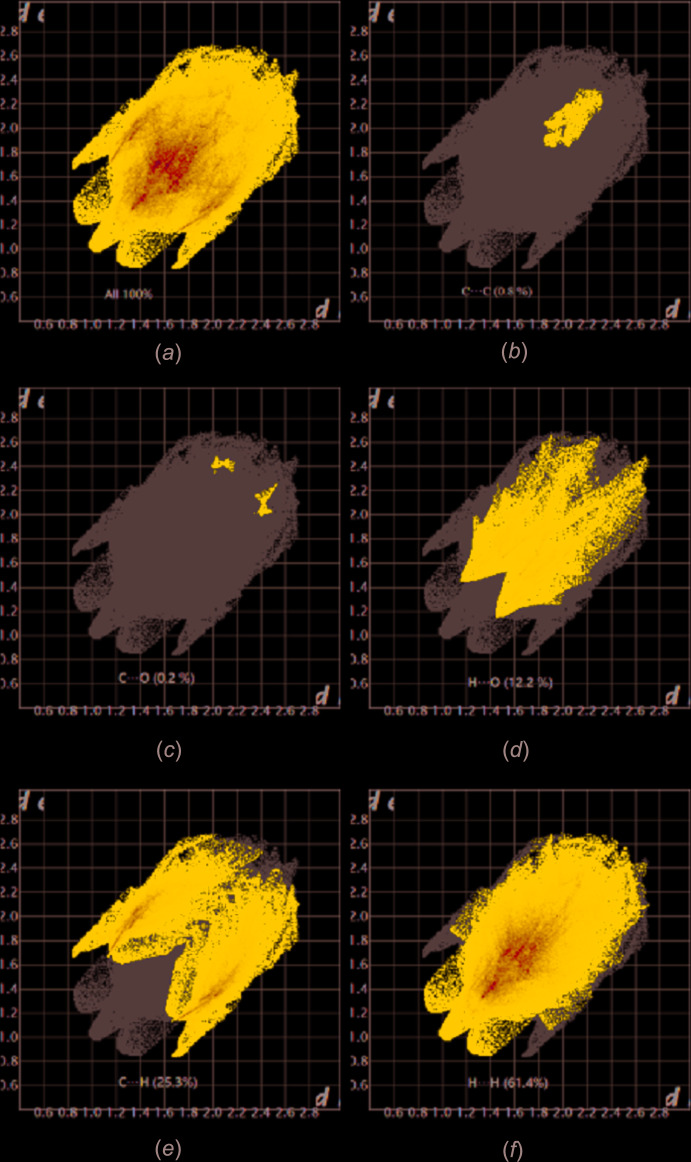
Two-dimensional fingerprint plots for the title compound: (*a*) all inter­molecular inter­actions, (*b*) C⋯C contacts, (*c*) C⋯O/O⋯C contacts, (*d*) H⋯O/O⋯H contacts, (*e*) C⋯H/H⋯C contacts and (*f*) H⋯H contacts.

In order to investigate the inter­molecular inter­actions in a visual manner, a Hirshfeld surface analysis was performed using *CrystalExplorer* (Spackman & Jayatilaka, 2009 ▸; Turner *et al.*, 2017 ▸). The bright-red spots on the Hirshfeld surface mapped over *d*_norm_ (Fig. 3 ▸ *a*) show the presence of C—H⋯π inter­actions with neighbouring mol­ecules. The absence of adjacent red and blue triangles on the shape-index map (Fig. 3 ▸ *b*) suggests that there are no notable π–π inter­actions. The fingerprint plots (Fig. 4 ▸) are given for all contacts, and those delineated into C⋯C (0.8%), C⋯O/O⋯C (0.2%), H⋯O/O⋯H (12.2%), C⋯H/H⋯C (25.3%) and H⋯H (61.4%) contacts. The most important contributions to the crystal packing are H⋯H and C⋯H/H⋯C contacts.

## Database survey   

A search of the Cambridge Structural Database (CSD, version 5.40, last update August 2019; Groom *et al.*, 2016 ▸) gave twelve hits for compounds having a 9,10-di­hydro-9,10-ep­oxy­anthracene fragment. In these structures, all five-membered rings are in an envelope conformation and the benzene rings in each fused ring system show a similar dihedral angle of *ca* 72°. A search for structures with a 3,6,7,8-tetra­hydro-1*H*-indeno­[4,5-*c*]furan fragment revealed two hits, and one of the compounds, dimethyl 5-phenyl-4-(phenyl­ethyn­yl)-1,3,6,9-tetra­hydro-2*H*-6,9-ep­oxy­cyclo­penta­[*a*]naphthalene-2,2-di­carb­oxyl­ate (refcode IKOJUP; Zhang *et al.*, 2015 ▸) is closely related to the title compound.

## Synthesis and crystallization   

Dimethyl 2,2-bis­[5-(4-ethyl­phen­yl)penta-2,4-diyn-1-yl]malonate (0.46 g) and 1,3-di­phenyl­isobenzo­furan (0.3 g) were added to toluene (2.0 ml), and the mixture was stirred at room temperature and then heated at 373 K for 10 h in air. The reaction mixture was cooled to room temperature, and the solvent was evaporated *in vacuo*. The residue was purified by column chromatography on silica gel using *n*-hexa­ne/ethyl acetate (20:1, *v*:*v*) as eluent to afford the compound (0.46 g) as a white solid. Part of the purified product was redissolved in *n*-hexa­ne/ethyl acetate and colourless crystals suitable for X-ray diffraction were formed after slow evaporation for several days.

Spectroscopic data: FT–IR (KBr): 3028, 2949, 1741, 1728, 1508, 1446, 1243, 1049, 619 cm^−1^; ^1^H NMR (C_6_D_6_, 300 MHz): δ 8.17–8.14 (*d*, *J* = 7.5Hz, 2H), 7.75–7.73 (*d*, *J* = 6.0 Hz, 1H), 7.60–7.56 (*m*, 3H), 7.35–7.31 (*m*, 2H), 7.24–7.16 (*m*, 5H), 7.00 (*s*, 2H), 6.91–6.76 (*m*, 7H), 4.29–4.23 (*d*, *J* = 17.1 Hz, 1H), 3.80–3.74 (*d*, *J* = 16.8Hz, 2H), 3.49–3.43 (*d*, *J* = 16.5Hz, 1H), 3.19 (*s*, 3H), 3.15 (*s*, 3H), 2.39–2.37 (*m*, 2H), 2.27–2.25 (*m*, 2H), 1.07–1.02 (*t*, *J* = 7.2Hz, 3H), 0.96–0.91 (*t*, *J* = 7.2Hz, 3H); ^13^C NMR (C_6_D_6_, 125 MHz): δ 171.9, 171.4, 151.6, 151.3, 149.6, 147.7, 144.6, 143.8, 143.1, 137.6, 135.9, 134.8, 134.7, 132.6, 131.9, 130.0, 129.8, 129.4, 128.9, 128.4, 127.7, 127.0, 126.4, 126.2, 122.9, 122.4, 121.3, 118.4, 97.3, 93.1, 91.1, 87.7, 60.8, 52.6, 52.5, 41.1, 39.7, 29.1, 29.1, 16.3, 15.6.

## Refinement   

Crystal data, data collection and structure refinement details are summarized in Table 2 ▸. All H atoms were included in calculated positions (C—H = 0.93–0.97 Å) using a riding model, with *U*_iso_(H) = 1.5 or 1.2*U*_eq_(C). The ethyl group (C46–C47) was found to be disordered over two sites around the C43—C46 bond axis and the occupancies were refined to 0.548 (9) and 0.452 (9). For the two ethyl groups (C36–C37 and C46—C47), displacement restraints (*DELU* and *SIMU*) were applied. For the disordered atoms (C47 and C47*A*), *ISOR* restraint and *EADP* constraint were also applied.

## Supplementary Material

Crystal structure: contains datablock(s) general, I. DOI: 10.1107/S2056989020006404/is5535sup1.cif

Structure factors: contains datablock(s) I. DOI: 10.1107/S2056989020006404/is5535Isup2.hkl

CCDC reference: 2003756

Additional supporting information:  crystallographic information; 3D view; checkCIF report

## Figures and Tables

**Table 1:** Hydrogen-bond geometry (Å, °) *Cg*4 and *Cg*7 are the centroids of the C3/C4/C13–C16 and C18–C23 rings, respectively.

*D*—H⋯*A*	*D*—H	H⋯*A*	*D*⋯*A*	*D*—H⋯*A*
C20—H20⋯*Cg*4^i^	0.93	2.61	3.516 (3)	165
C26—H26⋯*Cg*7^ii^	0.93	2.82	3.713 (3)	162

Symmetry codes: (i) 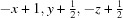; (ii) 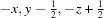.

**Table 2:** Experimental details

Crystal data	
Chemical formula	C_51_H_42_O_5_
*M*_r_	734.84
Crystal system, space group	Monoclinic, *P*2_1_/*c*
Temperature (K)	293
*a*, *b*, *c* (Å)	11.3654 (10), 14.2430 (12), 25.168 (2)
β (°)	91.207 (1)
*V* (Å^3^)	4073.2 (6)
*Z*	4
Radiation type	Mo *K*α
μ (mm^−1^)	0.08
Crystal size (mm)	0.23 × 0.22 × 0.20

Data collection	
Diffractometer	Bruker APEXII CCD area detector
Absorption correction	Multi-scan (*SADABS*; Bruker, 2002 ▸)
*T*_min_, *T*_max_	0.983, 0.985
No. of measured, independent and observed [*I* > 2σ(*I*)] reflections	34227, 9159, 6819
*R*_int_	0.031
(sin θ/λ)_max_ (Å^−1^)	0.650

Refinement	
*R*[*F* ^2^ > 2σ(*F* ^2^)], *wR*(*F* ^2^), *S*	0.056, 0.169, 1.04
No. of reflections	9159
No. of parameters	515
No. of restraints	20
H-atom treatment	H-atom parameters constrained
Δρ_max_, Δρ_min_ (e Å^−3^)	0.33, −0.30

Computer programs: *APEX2* and *SAINT* (Bruker, 2002 ▸), *SHLEXT* (Sheldrick, 2015*a* ▸), *SHELXL2018/3* (Sheldrick, 2015*b* ▸), *DIAMOND* (Brandenburg & Putz, 2012 ▸) and *OLEX2* (Dolomanov *et al.*, 2009 ▸).

## supplementary crystallographic information

### Crystal data

**Table:**

C_51_H_42_O_5_	*F*(000) = 1552
*M**_r_* = 734.84	*D*_x_ = 1.198 Mg m^−^^3^
Monoclinic, *P*2_1_/*c*	Mo *K*α radiation, λ = 0.71073 Å
*a* = 11.3654 (10) Å	Cell parameters from 9893 reflections
*b* = 14.2430 (12) Å	θ = 2.2–27.3°
*c* = 25.168 (2) Å	µ = 0.08 mm^−^^1^
β = 91.207 (1)°	*T* = 293 K
*V* = 4073.2 (6) Å^3^	Block, colourless
*Z* = 4	0.23 × 0.22 × 0.20 mm

### Data collection

**Table:**

Bruker APEXII CCD area detector diffractometer	6819 reflections with *I* > 2σ(*I*)
Graphite monochromator	*R*_int_ = 0.031
phi and ω scans	θ_max_ = 27.5°, θ_min_ = 1.6°
Absorption correction: multi-scan (SADABS; Bruker, 2002)	*h* = −14→14
*T*_min_ = 0.983, *T*_max_ = 0.985	*k* = −18→17
34227 measured reflections	*l* = −32→32
9159 independent reflections	

### Refinement

**Table:**

Refinement on *F*^2^	Hydrogen site location: inferred from neighbouring sites
Least-squares matrix: full	H-atom parameters constrained
*R*[*F*^2^ > 2σ(*F*^2^)] = 0.056	*w* = 1/[σ^2^(*F*_o_^2^) + (0.0848*P*)^2^ + 1.1068*P*] where *P* = (*F*_o_^2^ + 2*F*_c_^2^)/3
*wR*(*F*^2^) = 0.169	(Δ/σ)_max_ < 0.001
*S* = 1.04	Δρ_max_ = 0.33 e Å^−^^3^
9159 reflections	Δρ_min_ = −0.30 e Å^−^^3^
515 parameters	Extinction correction: SHELXL-2018/3, Fc^*^=kFc[1+0.001xFc^2^λ^3^/sin(2θ)]^-1/4^
20 restraints	Extinction coefficient: 0.0288 (14)
Primary atom site location: dual	

### Special details

**Table:**

Geometry. All esds (except the esd in the dihedral angle between two l.s. planes) are estimated using the full covariance matrix. The cell esds are taken into account individually in the estimation of esds in distances, angles and torsion angles; correlations between esds in cell parameters are only used when they are defined by crystal symmetry. An approximate (isotropic) treatment of cell esds is used for estimating esds involving l.s. planes.

### Fractional atomic coordinates and isotropic or equivalent isotropic displacement parameters (Å^2^)

**Table:**

	*x*	*y*	*z*	*U*_iso_*/*U*_eq_	Occ. (<1)
C1	0.67845 (14)	0.32574 (12)	0.38548 (7)	0.0473 (4)	
C2	0.59520 (14)	0.34546 (12)	0.33729 (7)	0.0457 (4)	
H2A	0.553476	0.404303	0.341554	0.055*	
H2B	0.638223	0.347262	0.304421	0.055*	
C3	0.51201 (13)	0.26312 (10)	0.33826 (6)	0.0387 (3)	
C4	0.40374 (13)	0.24906 (10)	0.31442 (6)	0.0371 (3)	
C5	0.32602 (13)	0.30966 (11)	0.27700 (6)	0.0393 (3)	
C6	0.33055 (15)	0.25996 (12)	0.22280 (6)	0.0441 (4)	
C7	0.38339 (19)	0.27862 (14)	0.17523 (7)	0.0605 (5)	
H7	0.425215	0.333947	0.170291	0.073*	
C8	0.3725 (2)	0.21236 (17)	0.13465 (8)	0.0737 (6)	
H8	0.405699	0.224808	0.101928	0.088*	
C9	0.3143 (2)	0.12947 (16)	0.14186 (8)	0.0692 (6)	
H9	0.309206	0.086263	0.114200	0.083*	
C10	0.26254 (17)	0.10908 (14)	0.19004 (7)	0.0550 (4)	
H10	0.224551	0.052124	0.195377	0.066*	
C11	0.26919 (14)	0.17587 (12)	0.22980 (6)	0.0441 (4)	
C12	0.22847 (13)	0.17681 (11)	0.28746 (6)	0.0407 (3)	
C13	0.34321 (13)	0.16415 (11)	0.32089 (6)	0.0379 (3)	
C14	0.39041 (14)	0.08984 (10)	0.34996 (6)	0.0392 (3)	
C15	0.50249 (14)	0.10493 (11)	0.37516 (6)	0.0402 (3)	
C16	0.55998 (13)	0.18993 (11)	0.36889 (6)	0.0405 (3)	
C17	0.68014 (15)	0.21714 (12)	0.38992 (7)	0.0505 (4)	
H17A	0.741600	0.190258	0.368502	0.061*	
H17B	0.691648	0.197196	0.426511	0.061*	
C18	0.33666 (13)	0.41416 (11)	0.28409 (6)	0.0409 (3)	
C19	0.3906 (2)	0.47302 (14)	0.24958 (9)	0.0707 (6)	
H19	0.419039	0.449371	0.217893	0.085*	
C20	0.4037 (2)	0.56778 (15)	0.26106 (11)	0.0796 (7)	
H20	0.442902	0.606349	0.237434	0.096*	
C21	0.3608 (2)	0.60460 (14)	0.30570 (9)	0.0666 (5)	
H21	0.370153	0.668137	0.313189	0.080*	
C22	0.3027 (3)	0.54696 (16)	0.34017 (9)	0.0839 (8)	
H22	0.271439	0.571660	0.371031	0.101*	
C23	0.2907 (2)	0.45260 (14)	0.32917 (8)	0.0722 (6)	
H23	0.250777	0.414320	0.352688	0.087*	
C24	0.11663 (14)	0.12878 (11)	0.30320 (7)	0.0449 (4)	
C25	0.03121 (16)	0.10294 (15)	0.26622 (8)	0.0594 (5)	
H25	0.044218	0.112279	0.230250	0.071*	
C26	−0.07348 (18)	0.06334 (17)	0.28205 (10)	0.0726 (6)	
H26	−0.129658	0.045456	0.256624	0.087*	
C27	−0.09501 (18)	0.05029 (16)	0.33468 (11)	0.0732 (6)	
H27	−0.165477	0.023556	0.345167	0.088*	
C28	−0.01173 (18)	0.07699 (16)	0.37203 (9)	0.0692 (6)	
H28	−0.026166	0.068634	0.407954	0.083*	
C29	0.09345 (16)	0.11620 (14)	0.35656 (8)	0.0553 (4)	
H29	0.149089	0.134284	0.382182	0.066*	
C30	0.32887 (14)	−0.00163 (11)	0.35447 (6)	0.0432 (4)	
C31	0.29777 (19)	−0.03780 (13)	0.40336 (8)	0.0589 (5)	
H31	0.322227	−0.007798	0.434503	0.071*	
C32	0.2301 (2)	−0.11886 (15)	0.40585 (9)	0.0715 (6)	
H32	0.209412	−0.142128	0.438890	0.086*	
C33	0.19276 (19)	−0.16572 (14)	0.36061 (10)	0.0663 (5)	
C34	0.22917 (18)	−0.13207 (14)	0.31229 (9)	0.0630 (5)	
H34	0.208653	−0.164396	0.281355	0.076*	
C35	0.29538 (16)	−0.05141 (12)	0.30907 (7)	0.0511 (4)	
H35	0.318123	−0.029833	0.275960	0.061*	
C36	0.1087 (3)	−0.24775 (19)	0.36355 (14)	0.1012 (8)	
H36A	0.130152	−0.294591	0.337497	0.121*	
H36B	0.115443	−0.276292	0.398479	0.121*	
C37	−0.0154 (3)	−0.2178 (2)	0.35341 (15)	0.1107 (8)	
H37A	−0.023092	−0.192468	0.318176	0.166*	
H37B	−0.036399	−0.170793	0.378823	0.166*	
H37C	−0.066614	−0.271027	0.356715	0.166*	
C38	0.55911 (15)	0.03226 (11)	0.40587 (6)	0.0452 (4)	
C39	0.60910 (16)	−0.02503 (12)	0.43218 (7)	0.0488 (4)	
C40	0.66926 (16)	−0.09620 (12)	0.46286 (6)	0.0479 (4)	
C41	0.60811 (19)	−0.15832 (14)	0.49438 (7)	0.0585 (5)	
H41	0.526864	−0.153142	0.496954	0.070*	
C42	0.6680 (2)	−0.22811 (15)	0.52203 (8)	0.0707 (6)	
H42	0.625907	−0.269003	0.543290	0.085*	
C43	0.7868 (2)	−0.23862 (16)	0.51901 (8)	0.0722 (6)	
C44	0.8472 (2)	−0.17530 (19)	0.48899 (10)	0.0843 (7)	
H44	0.928670	−0.179979	0.487244	0.101*	
C45	0.7898 (2)	−0.10488 (17)	0.46135 (9)	0.0740 (6)	
H45	0.833043	−0.062660	0.441415	0.089*	
C46	0.8509 (4)	−0.3181 (2)	0.54713 (12)	0.1143 (10)	
H46A	0.934866	−0.306750	0.545226	0.137*	0.548 (9)
H46B	0.830517	−0.316916	0.584359	0.137*	0.548 (9)
H46C	0.800764	−0.344924	0.573909	0.137*	0.452 (9)
H46D	0.921346	−0.294122	0.564826	0.137*	0.452 (9)
C47	0.8270 (10)	−0.4094 (5)	0.5268 (3)	0.1202 (12)	0.548 (9)
H47A	0.746809	−0.425897	0.533709	0.180*	0.548 (9)
H47B	0.878766	−0.453993	0.543778	0.180*	0.548 (9)
H47C	0.839107	−0.410076	0.489164	0.180*	0.548 (9)
C48	0.62644 (18)	0.37218 (14)	0.43477 (8)	0.0582 (5)	
C49	0.6058 (4)	0.3681 (3)	0.52714 (10)	0.1300 (14)	
H49A	0.525800	0.388312	0.522056	0.195*	
H49B	0.654441	0.421213	0.535929	0.195*	
H49C	0.610197	0.323156	0.555545	0.195*	
C50	0.80048 (16)	0.36611 (15)	0.37898 (8)	0.0586 (5)	
C51	0.96438 (18)	0.3759 (2)	0.32348 (11)	0.0918 (8)	
H51A	0.979822	0.369125	0.286322	0.138*	
H51B	1.018019	0.337334	0.343772	0.138*	
H51C	0.974645	0.440409	0.333676	0.138*	
O1	0.5728 (2)	0.44286 (14)	0.43391 (7)	0.1123 (7)	
O2	0.64680 (18)	0.32502 (13)	0.47847 (6)	0.0914 (6)	
O3	0.8505 (2)	0.4118 (2)	0.41081 (10)	0.1535 (12)	
O4	0.84501 (12)	0.34724 (14)	0.33351 (6)	0.0822 (5)	
O5	0.21155 (9)	0.27792 (7)	0.29414 (4)	0.0413 (3)	
C47A	0.8856 (12)	−0.3967 (6)	0.5060 (4)	0.1202 (12)	0.452 (9)
H47D	0.816351	−0.417779	0.487026	0.180*	0.452 (9)
H47E	0.921157	−0.448603	0.524583	0.180*	0.452 (9)
H47F	0.940502	−0.371375	0.481252	0.180*	0.452 (9)

### Atomic displacement parameters (Å^2^)

**Table:**

	*U*^11^	*U*^22^	*U*^33^	*U*^12^	*U*^13^	*U*^23^
C1	0.0418 (9)	0.0482 (9)	0.0518 (9)	−0.0037 (7)	−0.0007 (7)	0.0007 (7)
C2	0.0389 (8)	0.0445 (9)	0.0536 (9)	−0.0025 (7)	−0.0009 (7)	0.0047 (7)
C3	0.0372 (8)	0.0374 (8)	0.0416 (8)	0.0023 (6)	0.0049 (6)	0.0001 (6)
C4	0.0368 (8)	0.0365 (8)	0.0381 (7)	0.0044 (6)	0.0038 (6)	0.0024 (6)
C5	0.0347 (7)	0.0407 (8)	0.0425 (8)	0.0043 (6)	0.0033 (6)	0.0042 (6)
C6	0.0447 (9)	0.0460 (9)	0.0415 (8)	0.0102 (7)	0.0002 (6)	0.0053 (6)
C7	0.0756 (13)	0.0577 (11)	0.0487 (10)	0.0101 (9)	0.0119 (9)	0.0099 (8)
C8	0.1065 (18)	0.0739 (14)	0.0413 (10)	0.0202 (13)	0.0150 (10)	0.0041 (9)
C9	0.0949 (16)	0.0671 (13)	0.0454 (10)	0.0213 (12)	−0.0034 (10)	−0.0093 (9)
C10	0.0620 (11)	0.0530 (10)	0.0496 (9)	0.0114 (8)	−0.0087 (8)	−0.0058 (8)
C11	0.0428 (8)	0.0469 (9)	0.0422 (8)	0.0085 (7)	−0.0044 (6)	0.0011 (7)
C12	0.0398 (8)	0.0382 (8)	0.0439 (8)	0.0029 (6)	−0.0011 (6)	−0.0008 (6)
C13	0.0377 (8)	0.0390 (8)	0.0372 (7)	0.0009 (6)	0.0025 (6)	−0.0013 (6)
C14	0.0425 (8)	0.0374 (8)	0.0378 (7)	0.0016 (6)	0.0020 (6)	−0.0007 (6)
C15	0.0441 (8)	0.0383 (8)	0.0381 (7)	0.0046 (6)	−0.0002 (6)	−0.0002 (6)
C16	0.0384 (8)	0.0412 (8)	0.0419 (8)	0.0046 (6)	−0.0001 (6)	−0.0013 (6)
C17	0.0441 (9)	0.0487 (10)	0.0583 (10)	0.0032 (7)	−0.0075 (7)	0.0010 (8)
C18	0.0360 (8)	0.0404 (8)	0.0464 (8)	0.0050 (6)	−0.0014 (6)	0.0046 (6)
C19	0.0839 (15)	0.0505 (11)	0.0792 (14)	−0.0058 (10)	0.0384 (12)	0.0003 (10)
C20	0.0913 (17)	0.0488 (11)	0.1003 (17)	−0.0142 (11)	0.0370 (14)	0.0061 (11)
C21	0.0686 (13)	0.0428 (10)	0.0881 (15)	−0.0037 (9)	−0.0069 (11)	−0.0027 (10)
C22	0.136 (2)	0.0538 (12)	0.0624 (13)	−0.0023 (13)	0.0167 (13)	−0.0098 (10)
C23	0.1158 (19)	0.0472 (11)	0.0544 (11)	−0.0035 (11)	0.0213 (11)	0.0020 (8)
C24	0.0375 (8)	0.0409 (8)	0.0562 (9)	0.0011 (6)	−0.0006 (7)	−0.0015 (7)
C25	0.0463 (10)	0.0669 (12)	0.0648 (11)	−0.0043 (9)	−0.0063 (8)	−0.0006 (9)
C26	0.0451 (11)	0.0768 (14)	0.0952 (17)	−0.0115 (10)	−0.0112 (10)	−0.0073 (12)
C27	0.0465 (11)	0.0692 (14)	0.1044 (18)	−0.0115 (9)	0.0113 (11)	0.0022 (12)
C28	0.0558 (12)	0.0788 (14)	0.0735 (13)	−0.0082 (10)	0.0144 (10)	0.0057 (11)
C29	0.0460 (9)	0.0616 (11)	0.0584 (10)	−0.0047 (8)	0.0046 (8)	−0.0039 (8)
C30	0.0457 (9)	0.0370 (8)	0.0469 (8)	0.0007 (6)	−0.0015 (7)	0.0020 (6)
C31	0.0739 (13)	0.0520 (10)	0.0507 (10)	−0.0127 (9)	0.0008 (9)	0.0039 (8)
C32	0.0831 (15)	0.0606 (12)	0.0711 (13)	−0.0150 (11)	0.0081 (11)	0.0159 (10)
C33	0.0607 (12)	0.0447 (10)	0.0935 (15)	−0.0082 (9)	0.0002 (11)	0.0004 (10)
C34	0.0645 (12)	0.0487 (10)	0.0754 (13)	−0.0041 (9)	−0.0082 (10)	−0.0122 (9)
C35	0.0562 (10)	0.0447 (9)	0.0525 (9)	0.0020 (8)	−0.0017 (8)	−0.0033 (7)
C36	0.0879 (13)	0.0697 (14)	0.147 (2)	−0.0314 (12)	0.0153 (15)	−0.0122 (14)
C37	0.0869 (14)	0.0927 (16)	0.153 (2)	−0.0297 (13)	0.0180 (16)	−0.0223 (15)
C38	0.0502 (9)	0.0410 (9)	0.0442 (8)	0.0037 (7)	−0.0025 (7)	−0.0001 (7)
C39	0.0579 (10)	0.0417 (9)	0.0465 (9)	0.0031 (7)	−0.0051 (7)	0.0012 (7)
C40	0.0600 (11)	0.0440 (9)	0.0395 (8)	0.0065 (7)	−0.0037 (7)	0.0037 (7)
C41	0.0670 (12)	0.0549 (11)	0.0538 (10)	0.0038 (9)	0.0074 (9)	0.0054 (8)
C42	0.0971 (17)	0.0601 (12)	0.0553 (11)	0.0041 (11)	0.0121 (11)	0.0185 (9)
C43	0.0997 (18)	0.0679 (13)	0.0488 (10)	0.0251 (12)	−0.0048 (11)	0.0144 (9)
C44	0.0658 (14)	0.1027 (19)	0.0844 (16)	0.0242 (13)	−0.0013 (12)	0.0346 (14)
C45	0.0635 (13)	0.0812 (15)	0.0772 (14)	0.0060 (11)	0.0020 (10)	0.0357 (12)
C46	0.166 (3)	0.0848 (15)	0.0915 (18)	0.0494 (19)	−0.0128 (17)	0.0307 (13)
C47	0.172 (3)	0.0868 (17)	0.101 (2)	0.048 (2)	−0.006 (2)	0.0243 (18)
C48	0.0632 (12)	0.0553 (11)	0.0563 (11)	−0.0093 (9)	0.0046 (9)	−0.0017 (9)
C49	0.212 (4)	0.123 (3)	0.0559 (14)	−0.015 (3)	0.0250 (19)	−0.0133 (16)
C50	0.0432 (10)	0.0687 (12)	0.0635 (11)	−0.0078 (8)	−0.0047 (8)	0.0000 (9)
C51	0.0402 (11)	0.131 (2)	0.1043 (19)	0.0036 (12)	0.0124 (11)	0.0305 (17)
O1	0.170 (2)	0.0872 (13)	0.0810 (12)	0.0496 (13)	0.0309 (12)	−0.0040 (10)
O2	0.1366 (16)	0.0861 (12)	0.0514 (8)	0.0106 (11)	0.0029 (9)	0.0001 (8)
O3	0.0914 (14)	0.236 (3)	0.1336 (18)	−0.0870 (18)	0.0265 (13)	−0.0883 (19)
O4	0.0428 (7)	0.1282 (14)	0.0761 (10)	−0.0100 (8)	0.0102 (7)	−0.0020 (9)
O5	0.0343 (5)	0.0400 (6)	0.0496 (6)	0.0032 (4)	0.0037 (4)	0.0001 (5)
C47A	0.172 (3)	0.0868 (17)	0.101 (2)	0.048 (2)	−0.006 (2)	0.0243 (18)

### Geometric parameters (Å, º)

**Table:**

C1—C2	1.548 (2)	C28—C29	1.383 (3)
C1—C17	1.551 (2)	C29—H29	0.9300
C1—C48	1.535 (3)	C30—C31	1.387 (2)
C1—C50	1.513 (2)	C30—C35	1.391 (2)
C2—H2A	0.9700	C31—H31	0.9300
C2—H2B	0.9700	C31—C32	1.389 (3)
C2—C3	1.507 (2)	C32—H32	0.9300
C3—C4	1.372 (2)	C32—C33	1.379 (3)
C3—C16	1.400 (2)	C33—C34	1.379 (3)
C4—C5	1.542 (2)	C33—C36	1.512 (3)
C4—C13	1.403 (2)	C34—H34	0.9300
C5—C6	1.539 (2)	C34—C35	1.377 (3)
C5—C18	1.504 (2)	C35—H35	0.9300
C5—O5	1.4514 (18)	C36—H36A	0.9700
C6—C7	1.377 (2)	C36—H36B	0.9700
C6—C11	1.399 (2)	C36—C37	1.491 (4)
C7—H7	0.9300	C37—H37A	0.9600
C7—C8	1.394 (3)	C37—H37B	0.9600
C8—H8	0.9300	C37—H37C	0.9600
C8—C9	1.368 (3)	C38—C39	1.188 (2)
C9—H9	0.9300	C39—C40	1.438 (2)
C9—C10	1.389 (3)	C40—C41	1.385 (3)
C10—H10	0.9300	C40—C45	1.377 (3)
C10—C11	1.382 (2)	C41—H41	0.9300
C11—C12	1.532 (2)	C41—C42	1.384 (3)
C12—C13	1.548 (2)	C42—H42	0.9300
C12—C24	1.504 (2)	C42—C43	1.363 (3)
C12—O5	1.4631 (18)	C43—C44	1.370 (3)
C13—C14	1.388 (2)	C43—C46	1.514 (3)
C14—C15	1.427 (2)	C44—H44	0.9300
C14—C30	1.484 (2)	C44—C45	1.377 (3)
C15—C16	1.386 (2)	C45—H45	0.9300
C15—C38	1.436 (2)	C46—H46A	0.9700
C16—C17	1.505 (2)	C46—H46B	0.9700
C17—H17A	0.9700	C46—H46C	0.9700
C17—H17B	0.9700	C46—H46D	0.9700
C18—C19	1.362 (2)	C46—C47	1.422 (8)
C18—C23	1.373 (3)	C46—C47A	1.581 (10)
C19—H19	0.9300	C47—H47A	0.9600
C19—C20	1.388 (3)	C47—H47B	0.9600
C20—H20	0.9300	C47—H47C	0.9600
C20—C21	1.341 (3)	C48—O1	1.177 (3)
C21—H21	0.9300	C48—O2	1.305 (2)
C21—C22	1.373 (3)	C49—H49A	0.9600
C22—H22	0.9300	C49—H49B	0.9600
C22—C23	1.378 (3)	C49—H49C	0.9600
C23—H23	0.9300	C49—O2	1.455 (3)
C24—C25	1.381 (2)	C50—O3	1.170 (3)
C24—C29	1.386 (2)	C50—O4	1.290 (2)
C25—H25	0.9300	C51—H51A	0.9600
C25—C26	1.383 (3)	C51—H51B	0.9600
C26—H26	0.9300	C51—H51C	0.9600
C26—C27	1.365 (3)	C51—O4	1.444 (3)
C27—H27	0.9300	C47A—H47D	0.9600
C27—C28	1.374 (3)	C47A—H47E	0.9600
C28—H28	0.9300	C47A—H47F	0.9600
			
C2—C1—C17	104.11 (14)	C24—C29—H29	119.7
C48—C1—C2	108.39 (15)	C28—C29—C24	120.55 (18)
C48—C1—C17	112.08 (15)	C28—C29—H29	119.7
C50—C1—C2	113.12 (14)	C31—C30—C14	121.56 (15)
C50—C1—C17	112.13 (15)	C31—C30—C35	117.97 (16)
C50—C1—C48	107.04 (15)	C35—C30—C14	120.40 (15)
C1—C2—H2A	111.2	C30—C31—H31	120.0
C1—C2—H2B	111.2	C30—C31—C32	120.02 (18)
H2A—C2—H2B	109.1	C32—C31—H31	120.0
C3—C2—C1	102.67 (13)	C31—C32—H32	119.1
C3—C2—H2A	111.2	C33—C32—C31	121.7 (2)
C3—C2—H2B	111.2	C33—C32—H32	119.1
C4—C3—C2	131.57 (14)	C32—C33—C36	121.1 (2)
C4—C3—C16	117.85 (14)	C34—C33—C32	117.85 (18)
C16—C3—C2	110.57 (14)	C34—C33—C36	120.9 (2)
C3—C4—C5	133.03 (14)	C33—C34—H34	119.4
C3—C4—C13	120.84 (14)	C35—C34—C33	121.10 (19)
C13—C4—C5	106.11 (13)	C35—C34—H34	119.4
C6—C5—C4	104.75 (12)	C30—C35—H35	119.4
C18—C5—C4	115.97 (13)	C34—C35—C30	121.17 (18)
C18—C5—C6	123.82 (13)	C34—C35—H35	119.4
O5—C5—C4	98.61 (11)	C33—C36—H36A	109.3
O5—C5—C6	99.63 (12)	C33—C36—H36B	109.3
O5—C5—C18	110.08 (12)	H36A—C36—H36B	108.0
C7—C6—C5	134.88 (17)	C37—C36—C33	111.5 (2)
C7—C6—C11	120.18 (16)	C37—C36—H36A	109.3
C11—C6—C5	104.84 (13)	C37—C36—H36B	109.3
C6—C7—H7	120.9	C36—C37—H37A	109.5
C6—C7—C8	118.2 (2)	C36—C37—H37B	109.5
C8—C7—H7	120.9	C36—C37—H37C	109.5
C7—C8—H8	119.2	H37A—C37—H37B	109.5
C9—C8—C7	121.52 (19)	H37A—C37—H37C	109.5
C9—C8—H8	119.2	H37B—C37—H37C	109.5
C8—C9—H9	119.6	C39—C38—C15	177.18 (19)
C8—C9—C10	120.74 (19)	C38—C39—C40	178.44 (18)
C10—C9—H9	119.6	C41—C40—C39	121.27 (17)
C9—C10—H10	121.0	C45—C40—C39	120.69 (17)
C11—C10—C9	118.07 (19)	C45—C40—C41	118.03 (17)
C11—C10—H10	121.0	C40—C41—H41	120.0
C6—C11—C12	105.85 (13)	C42—C41—C40	119.97 (19)
C10—C11—C6	121.20 (16)	C42—C41—H41	120.0
C10—C11—C12	132.75 (16)	C41—C42—H42	119.1
C11—C12—C13	104.24 (12)	C43—C42—C41	121.9 (2)
C24—C12—C11	121.28 (14)	C43—C42—H42	119.1
C24—C12—C13	120.73 (13)	C42—C43—C44	117.85 (19)
O5—C12—C11	99.24 (12)	C42—C43—C46	121.6 (2)
O5—C12—C13	99.41 (11)	C44—C43—C46	120.6 (3)
O5—C12—C24	107.67 (12)	C43—C44—H44	119.3
C4—C13—C12	104.25 (12)	C43—C44—C45	121.4 (2)
C14—C13—C4	122.24 (14)	C45—C44—H44	119.3
C14—C13—C12	133.42 (14)	C40—C45—H45	119.6
C13—C14—C15	116.80 (14)	C44—C45—C40	120.8 (2)
C13—C14—C30	122.15 (14)	C44—C45—H45	119.6
C15—C14—C30	121.05 (14)	C43—C46—H46A	108.4
C14—C15—C38	121.08 (14)	C43—C46—H46B	108.4
C16—C15—C14	119.95 (14)	C43—C46—H46C	109.6
C16—C15—C38	118.94 (14)	C43—C46—H46D	109.6
C3—C16—C17	109.98 (14)	C43—C46—C47A	110.3 (3)
C15—C16—C3	122.27 (14)	H46A—C46—H46B	107.5
C15—C16—C17	127.68 (14)	H46C—C46—H46D	108.1
C1—C17—H17A	111.2	C47—C46—C43	115.4 (4)
C1—C17—H17B	111.2	C47—C46—H46A	108.4
C16—C17—C1	102.81 (13)	C47—C46—H46B	108.4
C16—C17—H17A	111.2	C47A—C46—H46C	109.6
C16—C17—H17B	111.2	C47A—C46—H46D	109.6
H17A—C17—H17B	109.1	C46—C47—H47A	109.5
C19—C18—C5	124.73 (15)	C46—C47—H47B	109.5
C19—C18—C23	117.76 (17)	C46—C47—H47C	109.5
C23—C18—C5	117.51 (15)	H47A—C47—H47B	109.5
C18—C19—H19	119.5	H47A—C47—H47C	109.5
C18—C19—C20	120.91 (19)	H47B—C47—H47C	109.5
C20—C19—H19	119.5	O1—C48—C1	124.19 (19)
C19—C20—H20	119.5	O1—C48—O2	122.6 (2)
C21—C20—C19	121.0 (2)	O2—C48—C1	113.25 (18)
C21—C20—H20	119.5	H49A—C49—H49B	109.5
C20—C21—H21	120.5	H49A—C49—H49C	109.5
C20—C21—C22	118.92 (19)	H49B—C49—H49C	109.5
C22—C21—H21	120.5	O2—C49—H49A	109.5
C21—C22—H22	119.9	O2—C49—H49B	109.5
C21—C22—C23	120.2 (2)	O2—C49—H49C	109.5
C23—C22—H22	119.9	O3—C50—C1	124.8 (2)
C18—C23—C22	121.1 (2)	O3—C50—O4	122.0 (2)
C18—C23—H23	119.4	O4—C50—C1	113.23 (16)
C22—C23—H23	119.4	H51A—C51—H51B	109.5
C25—C24—C12	122.00 (16)	H51A—C51—H51C	109.5
C25—C24—C29	118.26 (16)	H51B—C51—H51C	109.5
C29—C24—C12	119.55 (15)	O4—C51—H51A	109.5
C24—C25—H25	119.6	O4—C51—H51B	109.5
C24—C25—C26	120.8 (2)	O4—C51—H51C	109.5
C26—C25—H25	119.6	C48—O2—C49	116.0 (2)
C25—C26—H26	119.7	C50—O4—C51	118.93 (19)
C27—C26—C25	120.5 (2)	C5—O5—C12	98.72 (10)
C27—C26—H26	119.7	C46—C47A—H47D	109.5
C26—C27—H27	120.3	C46—C47A—H47E	109.5
C26—C27—C28	119.43 (19)	C46—C47A—H47F	109.5
C28—C27—H27	120.3	H47D—C47A—H47E	109.5
C27—C28—H28	119.8	H47D—C47A—H47F	109.5
C27—C28—C29	120.4 (2)	H47E—C47A—H47F	109.5
C29—C28—H28	119.8		

### Hydrogen-bond geometry (Å, º)

*Cg*4 and *Cg*7 are the centroids of the C3/C4/C13–C16 and C18–C23 rings, respectively.

**Table:**

*D*—H···*A*	*D*—H	H···*A*	*D*···*A*	*D*—H···*A*
C20—H20···*Cg*4^i^	0.93	2.61	3.516 (3)	165
C26—H26···*Cg*7^ii^	0.93	2.82	3.713 (3)	162

Symmetry codes: (i) −*x*+1, *y*+1/2, −*z*+1/2; (ii) −*x*, *y*−1/2, −*z*+1/2.
